# Editorial: Eyeblink Classical Conditioning in Psychiatric Conditions: Novel Uses for a Classic Paradigm

**DOI:** 10.3389/fpsyt.2017.00048

**Published:** 2017-03-27

**Authors:** Tracy L. Greer, Lucien T. Thompson

**Affiliations:** ^1^Department of Psychiatry, UT Southwestern Medical Center, Dallas, TX, USA; ^2^Aging & Memory Research, Behavioral and Brain Sciences, University of Texas at Dallas, Richardson, TX, USA

**Keywords:** schizophrenia, alcohol abuse, hippocampus, frontal cortex, cerebellum, post-traumatic stress disorder, autism, depression

**Editorial on the Research Topic**

**Eyeblink Classical Conditioning in Psychiatric Conditions: Novel Uses for a Classic Paradigm**

As one of the most basic forms of associative learning, eyeblink conditioning (EBC) is a model paradigm with unique utility in the assessment of complex behavioral disorders, including psychiatric disorders. Two major EBC paradigms utilized with human subjects are delay EBC [in which a conditioned stimulus (CS; e.g., an auditory tone) co-terminates with an unconditioned stimulus (US; e.g., a corneal airpuff)] and trace EBC [in which CS presentation is followed after a silent interstimulus interval (termed the “trace” interval by Pavlov) by the US, with no CS-US overlap in time]. The neural substrates of EBC in these paradigms are well delineated and include the cerebellum and anterior interpositus nucleus, the hippocampus, and prefrontal cortex. Variability in acquisition, discrimination, timing, sensitization, and/or extinction of classically conditioned eyeblink responses provides insight into the behavioral and neurobiological characteristics of a variety of psychiatric and neurological disorders.

In this Research Topic, Kent et al. review the existing literature on EBC studies in schizophrenia, perhaps the most studied psychiatric diagnostic group with respect to EBC to date. In their review of 15 studies, they report that decreased percent CRs (impaired learning) is the most robust and replicated finding in this population, with equivocal findings associated with CR timing. Importantly, differences between schizophrenic and healthy controls in percent CRs appear to be reflective of cerebellar abnormalities—supported by neuroimaging data and data from first-degree relatives—rather than confounding issues such as medication use. However, the authors highlight the importance of methodological consistency across EBC paradigms for accurate interpretation and synthesis of data across studies, an issue critical in the evaluation of any behavioral measure. Bolbecker et al. report data on delay EBC in schizophrenia using an analytic approach—hierarchical liner modeling—that has the advantages of less restrictive assumptions and inclusion of unequal variances in comparison to more traditionally employed analytic approaches, such as repeated measures analysis of variance. The authors did not find differences between schizophrenic patients and age-matched healthy controls in learning rate, but did observe group differences in time to maximal learning level and plateau of learning response, with schizophrenic patients exhibiting saturation in learning earlier and at a lower level in comparison to healthy controls.

Cheng et al. provide summaries of both animal and human work describing the impact of alcohol use disorders in adults and fetal alcohol syndrome in children. EBC studies in these populations have illuminated structural and functional differences in both the mature and developing cerebellum, as well as learning differences in these diagnostic groups when compared to healthy controls.

Weiss and Disterhoft emphasize the utility of rabbit EBC for assessing cerebro-cerebellar functional connectivity. The role of hippocampus, fronto-temporal and cerebellar cortices, and of cerebellar deep nuclei has been well characterized in a wide variety of normal states as well as dysfunctional psychiatric conditions. Their review concentrates on models of schizophrenia and Alzheimer’s dementia, but also points out applications for normal aging, Parkinson’s disease, progressive supranuclear palsy, alcoholism, and post-traumatic stress disorder (PTSD). Indeed, the rabbit EBC model was developed in parallel with assessment of human EBC in a series of studies from the laboratories of Gormezano, his students, and colleagues, which have allowed mechanistic, physiological, and pharmacological assessments to be carried out in depth.

Schreurs and Burhans detail development of a preclinical EBC extinction model with potential utility for treatment of inappropriate conditioned behaviors and hyperarousal in individuals suffering from PTSD, whether combat veterans or approximately 5–25% of the general populace who experience severe anxiety, sleeplessness, hypervigilance, and/or flashbacks after trauma constitute a group at extreme risk for suicide. Their exposure paradigm uses conditioning-specific reflex modification and attenuated intensity US presentations to enhance the likely clinical utility for human applications.

Janke et al. discuss dysregulation of brain-derived neurotrophic factor (BDNF) in anxiety disorders, including impaired behavioral inhibition temperament (BI). They describe facilitated delay EBC in both human and animal subjects exhibiting BI. In their animal model after EBC, increases in mRNA for hippocampal BDNF were observed in both the dentate gyrus and CA3 regions, along with upregulation of TrkB receptors and downstream activity-related cytoskeletal protein (Arc) in the hippocampus, but these increases were blunted in the strain showing faster acquisition and hyper-sensitivity to both CS and US. Hippocampal BDNF administration reversed the behavioral sensitization, suggesting treatment strategies that enhance that hippocampal BDNF may be effective for a variety of anxiety disorders.

Welsh and Oristaglio provide a secondary analysis of children with autism spectrum disorder (ASD) who underwent both delay and trace EBC. They grouped children based on their diagnosis into either an autistic disorder group or an Asperger’s syndrome or Pervasive Developmental Disorder (Asp/PDD) group. Neither ASD group showed differences in CR acquisition compared to an age- and IQ-matched group of typically developing (TD) children. However, the groups differed with respect to CR timing alterations, with the Asp/PDD group showing delayed CR onset and peak latencies during trace conditioning that were not observed in the ASD or TD groups. These data may illustrate differences in the underlying biology of these two diagnostic groups that are expressed as behavioral differences. Although the authors indicate that these differences must be further tested in a larger trial, these data are representative of the types of approaches that are supported by the NIMH’s Research Domain Criterion (RDoc) initiative that aims to use a variety of approaches, including behavioral phenotyping, to help our field better distinguish brain dysfunction among patients with psychiatric illnesses.

Similarly, Parker describes the potential for timing tasks to aid in our understanding of cognitive impairment across diagnostic groups, again in line with the RDoC approach. She asserts that tasks that involve temporal processing, such as EBC, can reflect connectivity between the cerebellum and frontal cortex, and in particular, hypothesizes that the medial frontal cortex plays a critical role in cognitive processing that occurs in timing tasks. This reciprocal relationship between cerebellar and frontal cortical regions is an important area of further investigation.

Cicchese and Berry also focus on timing and the critical role of theta (the 3–7 Hz EEG bandwidth prominent in medial temporal lobe) in many forms of learning and memory. They review non-theta contingent EBC, demonstrating its importance as a model system for characterizing neurobiological dysfunction in severe cognitive disorders, including schizophrenia, major depression, and Alzheimer’s disease. From their animal studies and an extensive review of the human literature, they argue that theta rhythms serve to coordinate timing and synchrony of activity in widely distributed brain systems critical for acquiring, consolidating, and retrieving memories, both for complex cognitive sequences and for relatively low-level tasks such as classical EBC.

The studies in this Research Topic highlight the utility of EBC in assessing the integrity of cerebellar and medial temporal lobe function in both normal and pathological states and support wider uses of this behavioral paradigm across a multitude of psychiatric disorders. The editors’ earlier work on impaired trace EBC in depressed individuals (1) illustrates qualitative diagnostic potential for this simple associative learning paradigm in future clinical practice. Increased familiarity of clinical practitioners with the straightforward methodology required, and adoption of standards for EBC testing and adjunct assessments of clinical characteristics of populations studied, should still further increase the use of this classical conditioning paradigm in diagnostic and treatment settings.

## Author Contributions

All authors listed have made substantial, direct, and intellectual contribution to the work and approved it for publication.

## Conflict of Interest Statement

The authors declare that the research was conducted in the absence of any commercial or financial relationships that could be construed as a potential conflict of interest.
